# Ischemia-Reperfusion Injuries Assessment during Pancreas Preservation

**DOI:** 10.3390/ijms22105172

**Published:** 2021-05-13

**Authors:** Thomas Prudhomme, John F. Mulvey, Liam A. J. Young, Benoit Mesnard, Maria Letizia Lo Faro, Ann Etohan Ogbemudia, Fungai Dengu, Peter J. Friend, Rutger Ploeg, James P. Hunter, Julien Branchereau

**Affiliations:** 1 Institut de Transplantation Urologie Néphrologie (ITUN), CHU Nantes, 44000 Nantes, France; prudhomme-thomas@hotmail.fr (T.P.); benoit.mesnard@aliceadsl.fr (B.M.); 2 Centre de Recherche en Transplantation et Immunologie (CRTI) UMR1064, INSERM, Université de Nantes, 44000 Nantes, France; 3 Nuffield Department of Surgical Sciences, University of Oxford, Oxford OX3 9DU, UK; john.mulvey@nds.ox.ac.uk (J.F.M.); liam.young@rdm.ox.ac.uk (L.A.J.Y.); letizia.lofaro@nds.ox.ac.uk (M.L.L.F.); ann.ogbemudia@nds.ox.ac.uk (A.E.O.); fungai.dengu@nds.ox.ac.uk (F.D.); peter.friend@nds.ox.ac.uk (P.J.F.); rutger.ploeg@nds.ox.ac.uk (R.P.); james.hunter@nds.ox.ac.uk (J.P.H.); 4 Oxford Centre for Clinical Magnetic Resonance Research (OCMR), Radcliffe Department of Medicine, University of Oxford, Oxford OX3 9DU, UK

**Keywords:** Ischemia Reperfusion, Pancreas perfusion, Hypothermic perfusion, Normothermic perfusion

## Abstract

Maintaining organ viability between donation and transplantation is of critical importance for optimal graft function and survival. To date in pancreas transplantation, static cold storage (SCS) is the most widely practiced method of organ preservation. The first experiments in ex vivo perfusion of the pancreas were performed at the beginning of the 20th century. These perfusions led to organ oedema, hemorrhage, and venous congestion after revascularization. Despite these early hurdles, a number of factors now favor the use of perfusion during preservation: the encouraging results of HMP in kidney transplantation, the development of new perfusion solutions, and the development of organ perfusion machines for the lung, heart, kidneys and liver. This has led to a resurgence of research in machine perfusion for whole organ pancreas preservation. This review highlights the ischemia-reperfusion injuries assessment during ex vivo pancreas perfusion, both for assessment in pre-clinical experimental models as well for future use in the clinic. We evaluated perfusion dynamics, oedema assessment, especially by impedance analysis and MRI, whole organ oxygen consumption, tissue oxygen tension, metabolite concentrations in tissue and perfusate, mitochondrial respiration, cell death, especially by histology, total cell free DNA, caspase activation, and exocrine and endocrine assessment.

## 1. Introduction

Maintaining organ viability between donation and transplantation is of critical importance for optimal graft function and survival. To date in pancreas transplantation, static cold storage (SCS) is the most widely practiced method of organ preservation. It is well known that the pancreas is highly susceptible to oedema and ischemia-reperfusion injury during both organ retrieval and preservation, which leads to damaging effects on the graft microvasculature [1] as well as dysfunction of both the endocrine and exocrine pancreas [2]. During SCS, the lack of oxygen as a terminal electron receptor for the mitochondrial electron transport chain precludes continued aerobic respiration, resulting in an inability of cells to generate sufficient adenosine triphosphate (ATP) for continued cellular function. In addition to cell death during ischemia, reperfusion itself exacerbates graft injury, with the islets of Langerhans being particularly susceptible. Since these are necessary for improved glucose control in the transplant recipient, this is associated with poor clinical outcomes after both islet [3] and whole pancreas transplantation [4]. Microvascular dysfunction is also a key contributor to the sequela of graft thrombosis, pancreatitis and eventual graft failure: a particular problem in pancreas transplantation compared with other abdominal organs [5].

Hypothermic machine perfusion (HMP) was first developed by Alexis Carrel in experimental transplant models over a century ago [6]. Belzer et al. subsequently introduced this procedure in the clinical setting of kidney transplantation [7], and since then HMP has grown in its application. The earliest published experimental ex vivo perfusions of pancreases were described in 1926 in a canine model by Babkin and Starling [8]. Florack et al. in 1983 performed autotransplantation after hypothermic perfusion of canine segmental pancreases using Ringer’s solution with a 30 mmHg perfusion pressure, by anastomosing the splenic pedicle to the iliac vessels [9,10]. However, all perfused pancreases demonstrated oedema, hemorrhage, and venous congestion after revascularization. Due to these poor results, there was a decline in research on whole pancreas perfusion for transplantation with a subsequent shift in focus towards islet isolation after HMP [11,12,13,14]. Despite these early hurdles, a number of factors now favor the use of perfusion during preservation: the encouraging results of HMP in kidney transplantation, the development of new perfusion solutions and the development of organ perfusion machines for the lung, heart, kidneys and liver. This has led to a resurgence of research in machine perfusion for whole organ pancreas preservation. The advantages of perfusion are twofold: first, it allows for potentially better preservation of the pancreas during organ storage, as has been shown for the liver [15] and the kidney [16,17,18]. Secondly, organ perfusion allows for organ function to be assessed prior to transplantation [19,20].

Herein, we define the elements of pancreas assessment during ex vivo machine perfusion, both for assessment in pre-clinical experimental models as well as for future use in the clinic.

## 2. Perfusion Dynamics by Non-Invasive Techniques

Ischemia-reperfusion injury leads to the activation of inflammatory pathways, the production of radical oxygen species, and the activation of vasoactive compounds. Combined, these result in endothelial cell damage and increased vascular resistance [21]. The triad of flow, perfusion pressure and vascular resistance evaluated during perfusion could therefore be a useful indicator of viability. The pancreas has complex vascular anatomy without the end arteries found in other established ex vivo perfused abdominal organs such as the liver and kidney. This means that in vivo, the pancreas is a low flow organ and so a high perfusion pressure, such as that used in the liver and kidney, causes endothelial injury and oedema to the pancreas. Conversely, if the pressure is too low, perfusate flow will not be adequate for efficacious perfusion.

Depending on the method of perfusion (e.g., hypothermic ex vivo perfusion, normothermic ex vivo perfusion, normothermic regional perfusion), pressures will vary differently due to changes in vascular resistance with temperature.

### 2.1. Hypothermic Ex Vivo Perfusion

In most of the experimental hypothermic perfusion studies (commonly conducted at 4 °C), pancreas perfusion is pressure-controlled with arterial pressures maintained at reference values. Thus, depending on intravascular resistance—which tends to be raised in damaged organs—arterial blood flow will change with constant perfusion pressure. Consequently, the flow, pressure, and resistance should be assessed and reported simultaneously.

Compared with continuous flow, HMP with pulsatile flow maintains the vascular bed and protects the endothelium [22]. Pulsatile flow may activate endothelial protective genes such as Kruppel-like factor 2 [23]. Kruppel-like factor 2 is thought to inhibit pro-inflammatory responses and protect the vascular endothelium [24,25], which Fukae et al. [25] showed to be mediated via the reduction in sympathetic nerve activity. This results in a reduction in vascular resistance, which may improve the microcirculation and therefore organ function.

### 2.2. Normothermic Ex Vivo Perfusion

Barlow et al. [26] and Nassar et al. [27] initially demonstrated the feasibility of ex vivo pancreas normothermic machine perfusion (NMP; 35–37 °C) in which they correlated amylase levels with both fat infiltration of the organ and exocrine function. Barlow et al. [26] used a pediatric cardiopulmonary bypass device with an organ chamber, venous reservoir, centrifugal blood pump and a heat exchanger to perfuse human pancreases unsuitable for transplantation. The systolic pressure used was 50–55 mmHg, lower than the systolic pressure usually used in kidney NMP [19,28]. Likewise, Nassar et al. [27] used a systolic pressure of 60 mmHg.

### 2.3. Normothermic Regional Perfusion and Flush

Normothermic regional perfusion (NRP) is the only preservation technique other than SCS that has been used in clinical pancreas transplantation. It is an extracorporeal membrane oxygenation-based technology that is currently performed in DCD donors. It involves cannulating the arterial and venous circulation to include only the abdominal organs, and then continuously perfusing them with oxygenated, normothermic blood. This emerging strategy in organ preservation has shown acceptable transplantation results for liver and variable results in kidneys in uncontrolled and controlled DCD donors [29,30,31]. Only three cases of successful solid organ pancreas transplantation and one islet cell transplant after NRP are reported but without data on outcomes [31,32]. Branchereau et al. [33] reported a preclinical evaluation of histological lesions in pancreas after NRP. They showed that flushing organs with an IGL1 preservation solution with a flow ≤500 mL/min did not affect pancreas histology.

## 3. Oedema Assessment

### 3.1. Non-Invasive Techniques

#### 3.1.1. Macroscopic Appearance

The macroscopic appearance of the pancreas is an important quality assessment tool that is used both formally and informally by transplant surgeons. Kuan et al. [34] developed the oedema scale (ES) in order to evaluate macroscopic oedema in hypothermic pancreas perfusion experimental studies (ES: 0 = none, 1 = mild, 2 = moderate, 3 = severe). This scale is used by several laboratories [14,35,36,37]. However, assessment remains subjective.

#### 3.1.2. Weight

The weight of the duodeno-pancreatic bloc is, in our mind, not a helpful evaluation criterion due to the presence of perfusion liquid in the duodenum. Thus, the total weight depends upon both the length of the duodenum preserved with the transplant and the rate of pancreatic secretion rather than purely the extent of oedema.

#### 3.1.3. Impedance Analysis

An alternative, non-invasive measure of tissue composition could be achieved using impedance analysis. This could be carried out during pancreas perfusion and is non-invasive, inexpensive and relatively simple [38]. To date, this not been used for the evaluation of pancreatic oedema during perfusion. Its development would allow better objectivity in the evaluation of pancreatic oedema in real time. We note, however, that impedance will be dependent upon the electrolytic composition of the perfusion solution.

### 3.2. Invasive Techniques

#### Wet-to-Dry Weight

A comparison of wet-to-dry weight is commonly used to normalise for variations in tissue water content, which Leemkuil et al. [14] proposed for objective evaluation of pancreatic oedema using tissue biopsies. The weight of the biopsy is measured immediately following collection (wet weight) and after the biopsy is dried in a heat block at 95° for 24 h to remove intra- and extracellular water (dry weight). Whilst this correlates directly with the oedema, it is both invasive and cannot be measured in real time.

## 4. Temperature by Non-Invasive Techniques

Assessment of the deep tissue and surrounding perfusate temperature is of paramount importance to indicate adequate normothermia or hypothermia settings.

In their experimental study, Dumciene and Supaviciene developed a transducer for non-invasive temperature assessment in deeper tissues [39]. Pouch et al. developed a B-mode ultrasound technology to assess deep tissue temperature [40] and Stauffer et al. developed a microwave radiometry technology [41]. Currently, these new technologies have only been described for experimental purposes.

## 5. pH by Non-Invasive Techniques

pH is commonly assessed in perfusate by blood gas analysis, with this measurement, depending upon the analyzer used, often being conducted at 37 °C. Since the ionization of water is an endothermic process, pH decreases (i.e., [H^+^] increases) with increases in temperature, as predicted by Le Chatilier’s principle [42]. Whilst measuring pH at 37 °C aids in the physiological interpretation of pH values (since the point of neutrality varies with temperature), it is important to note that this is not the actual pH in hypothermic tissue. In a clinical setting, for assessing perfusate pH, we should aim to maintain the pH gradient across the plasma membrane that is found in normal physiological conditions. Thus, in normothermic perfusion, the objective is to maintain a pH between 7.35 and 7.45. Thus, variations above or below these values are markers of pancreatic injuries.

## 6. Metabolic Status

### 6.1. Invasive Techniques

#### 6.1.1. High Energy Adenine Nucleotides

Adenosine triphosphate (ATP) content is a direct measure of cellular bioenergetics and has long been proposed as a diagnostic marker since the seminal studies of ischemia-reperfusion in the heart by Jennings and colleagues [43]. During ischemia, ATP levels are depleted due to a combination of reduced synthesis and ATP hydrolysis from uncoupled mitochondria. ATP hydrolysis results in ADP and inorganic phosphate (P_i_). The high-energy phosphate of ADP can be further utilized by adenylate kinase to produce ATP and AMP, with the latter further degraded to produce adenosine and subsequently the breakdown products inosine, xanthine and hypoxanthine. Metabolic recovery is generally not regarded as possible when the adenine nucleotide pool has been depleted, whereas tissue is salvageable if most of the adenine nucleotide pools remains [44]. ATP content has been used to assess the viability of the pancreas during perfusion [14,45,46]. ATP is commonly measured either in tissue following biopsy, either by UV-vis detection following high-performance liquid chromatography or by luminescence detection, commonly measuring the production of light by the conversion of luciferin to oxyluciferin catalysed by a luciferase. The latter also allows the measurement of ADP content following its conversion to ATP by exogenous adenylate kinase.

#### 6.1.2. Tissue Metabolite Concentrations

Ischemia is fundamentally a metabolic issue driven by the mismatch of oxygen supply and the minimum demand for tissue viability. This has led to considerable research regarding metabolism during ischemia and reperfusion. Indeed, tissue succinate has recently been demonstrated to be a universal marker for ischemia, conserved across both multiple organs [47] and species [48]. Upon reperfusion of ischemic tissue, this pool of succinate is rapidly metabolized to drive the opening of the mitochondrial permeability transition pore [49]. However, to date, no data are available regarding the concentration of succinate or other ischemia-related metabolites in the pancreas.

There are a variety of methods to measure tissue metabolite abundance. Tissue biopsies may be taken for analysis by mass spectrometry, which allows the profiling of a large range of metabolites. Depending on the details of the method used (extraction, separation chemistry, ionization method), these can assess a wide range of different chemistries from aqueous metabolites to lipids and metabolite identification performed in either a targeted or untargeted manner. However, they are destructive and therefore undesirable for use in the clinic given the reluctance to biopsy the pancreas.

#### 6.1.3. Mitochondrial Respiration

The rate of oxygen consumption is an important measure of mitochondrial function in all aerobic cells [50]. Due to problems with insufficient oxygen diffusion in tissue biopsies that artificially limit respiratory function, traditionally this has been performed first by isolating mitochondria by differential centrifugation. Respiratory capacity in these isolated organelles is then assessed by measuring oxygen consumption with a Clark type electrode [51]. This can be done in the presence of different mitochondrial substrates and/or inhibitors in order to infer further information.

The development of high-resolution apparatus to measure oxygen consumption has facilitated the use of smaller quantities of biological material. The Gnaiger lab reported the use of tissue biopsies weighing 2–7 mg from liver biopsies during preservation [52]. Whilst assessment must still take place in an exogenous environment, this removes the time delay needed for traditional mitochondrial isolation strategies. An alternative approach is to use methods for mitochondrial isolation that are much quicker, such as those reported by Zuurendonk and Tager [53].

#### 6.1.4. Tissue Oxygen Tension

The measurement of oxygen tension in perfusate is indicative of the actual oxygen tension in pancreas tissue [54]. No direct assessment of oxygen tension has been published to date in the context of pancreatic transplantation, and we thus examine the published literature from other fields.

Historically, this has been done using Clark-type electrodes, often contained within a thin (~300 µM diameter) needle. Oxygen at the cathode is reduced to form water, and the current measured at an applied voltage. The principal disadvantage of these systems is that they are therefore oxygen-consuming, and so without high levels of tissue perfusion will report a continuous decrease in oxygen tension over time, leading to an underestimation of pO_2_. This also means that taking repeat measurements is problematic.

These challenges led to the development of dynamic fluorescence quenching using fibreoptic probes [55,56,57]. An optical fibre carries light pulses which excite a ruthenium-based chromophore that is incorporated into the probe tip. Thus, the fluorescence signal is inversely proportional to the oxygen tension at the probe tip since. In the presence of oxygen, fluorescence is quenched by the nonradiative transfer of energy from the chromophore to molecular oxygen [58]. Unlike electrode-based technologies, this does not consume oxygen, making it more suitable to leave in place for continuous monitoring. In fact, measurement accuracy actually increases at low oxygen levels because of the longer decay time of the fluorescence signal. We note that despite their very small size (~250 µm), the introduction of any probe into the parenchyma could result in damage to the pancreas, with a theoretical risk of pancreatic fistula in the event of clinical use.

There are also a few methods that are less commonly applied by which oxygen tension can be estimated indirectly. The Balaban laboratory, for example, developed a system to assess the redox status of endogenous mitochondrial chromophores in the isolated perfused heart [59]. By using colour deconvolution, it is possible to measure the redox status of cytochrome c oxidase in the electron transport chain, which, due to its function of reducing oxygen to form water, therefore corresponds with mitochondrial oxygen tension. Other invasive techniques also exist to assess tissue hypoxia, such as nitroimidazole-based hypoxia markers [60] or mass spectrometry with an organ sample [61], and are available for live assessment [62]. We note that if destructive, post hoc methods are to be used, it would make more sense to directly measure relevant downstream species such as mitochondrial reactive oxygen species such as superoxides [63] that have a causative role in ischemia-reperfusion pathology rather than indirectly estimating oxygen tension.

### 6.2. Non-Invasive Techniques

#### 6.2.1. Whole Organ Oxygen Consumption

Whole graft oxygen consumption can be calculated non-invasively by the Fick principle [64,65,66]. Both arterial (Pa0_2_) and venous (Pv0_2_) oxygen tension can be assessed by blood gas analysis or continuously assess by endovascular probes. Combined with the solubility of oxygen in perfusate at a given temperature (a), this gives the oxygen content in perfusate entering or leaving the graft. Thus, the device was calibrated to the temperature. Perfusate flow rate (Q) should be indicated by the perfusion machine and can then be used to measure oxygen flux, which should then be expressed relative to the mass (m) of the pancreas. Oxygen consumption rate (OCR) is thus calculated as follows: OCR = (ΔP0_2_ × a × Q)/m. In HMP, the most common rate of perfusion was 15–20 mmHg [14,35,67], while in NMP, the rate was higher (50–60 mmHg) [26,27]. Thus, it was important to evaluate the variation in oxygen consumption rate and not just the gross value.

#### 6.2.2. Perfusate Metabolite Concentrations

Lactate in the systemic circulation has long been used as a correlate of skeletal muscle metabolic status, and the same principle can be used to assess organ metabolic status during machine prefusion. The interpretation is aided by the fact that no other organs are present during ex vivo perfusion. By assessing the concentration of metabolites in the perfusate as it enters and leaves the pancreas, we are able to calculate the flux. Lindeman and colleagues measured metabolite flux by arterial–venous differences in the context of kidney preservation, which appears to provide additional insight compared with tissue metabolite abundance [68].

A greater understanding of metabolic function during normal physiology (including both the fed and fasted states) will allow comparison with metabolite levels during perfusion. The Rabinowitz laboratory presented data on arterial–venous differences in anaesthetized pigs [69]. They reported significant heterogeneity between different abdominal organs. Specifically, in the case of the pancreas, the main metabolites consumed are amino acids (consistent with its exocrine role requiring significant protein synthesis for enzyme release), and there is unexpectedly a net release of TCA cycle intermediates. This was recapitulated in the mouse using stable isotope labelled substrates. To what extent this is also the case during machine perfusion of the pancreas is unknown, but one would expect better functioning organs to be closer to normal physiology.

#### 6.2.3. Tissue Oxygen Tension

Electron paramagnetic resonance (EPR) spectroscopy can also be used, but this analogously requires the injection of paramagnetic oxygen-sensitive material [70]. Currently, there is significant toxicity associated with EPR spin probes such as trityl that precludes their use in organs before human transplantation [71]. Nevertheless, isolated organ perfusion facilitates the use of higher-strength magnets compared with the in situ organ such that it is likely to be possible to use appropriately low probe levels in the near future.

## 7. Cell Death Assessment

### 7.1. Invasive Techniques

#### 7.1.1. Cellular Architecture by Histology

Hematoxylin and eosin (HE) staining allows evaluation of the effects of perfusion on the exocrine pancreas such as acinar necrosis and steatonecrosis. We recently published a classification of these lesions [67]. Interstitial oedema corresponds to an inter acinar oedema that begins at the periphery of the lobules and precedes necrosis (0: no in no inter-acinar enlargements; 1: slight interacinar perilobular enlargements; 2: moderate peri- and centrilobular interacinar enlargements; 3: marked and diffuse inter-acinar enlargements). Acinar necrosis evaluation represents ischemic necrosis of acini cells with cytoplasmic and nuclear alterations (0: none; 1: focal peri-lobular necrosis; 2: peri-lobular multifocal necrotic areas; 3: range of focal centrolobular or multifocal necrosis). Steatonecrosis evaluation corresponds to the degree of ischemic necrosis of adipocytes (0: none; 1: focal length; 2: plurifocal; 3: diffuse).

#### 7.1.2. Ultrastructure

Electron microscopy allows the evaluation of the vascular microarchitecture and pancreatic extracellular matrix changes throughout the perfusion [72,73]. Moreover, electron microscopy would allow a better characterization of beta cell function during perfusion [74].

#### 7.1.3. Total Cell Free DNA (cfDNA)

Upon cell death and rupture, cytosolic proteins, nuclear and mitochondrial DNA are released into the extracellular fluid where they circulate at a low concentration. Isolated organ perfusion circuits provide a number of advantages here. First, we know that the cfDNA must originate from the pancreatic graft rather than it being of unknown origin. Secondly, with the perfusate exposed to a smaller volume of biological tissue than the blood is in vivo, we expect the circulating half live of cfDNA to be extended, which therefore increases our power to detect it at low abundance.

cfDNA may be isolated (by one of a range of commercially available kits [75]) and then quantified. A variety of methods exist to do this: most simply fluorescence detection by UV-vis spectroscopy, though we note that a number of isolation strategies involve the use of a carrier RNA that will quantitatively dominate this signal, rendering it incompatible. More robustly, quantification can be performed by either conventional or digital qPCR with a suitable set of primers for a gene with ubiquitous expression in the graft.

Some authors have defined an integrity index for cfDNA [76]. This is generally calculated as a ratio between a longer DNA fragment in comparison to a shorter one [77]. During necrotic cell death, DNA fragments are produced seemingly at random, resulting in larger fragments than the shorter 180–200 bp fragments (or multiple thereof) that are produced during apoptosis due to cleavage between histones. This has yet to be reported during pancreas perfusion.

#### 7.1.4. Caspase Activation

Activated caspase-3 is at the convergence of the intrinsic and extrinsic apoptotic pathways and is the main executioner of apoptosis [78]. Both immunohistochemical analysis or assessment in tissue homogenates of cleaved caspase-3 are therefore indicative of the extent of apoptotic cell death during perfusion. We previously reported that endothelial cells on the outskirts of the islets are sensitive to cleaved caspase-3 immunohistochemistry [36].

### 7.2. Non-Invasive Techniques

#### Lactate Dehydrogenase (LDH)

Lactate dehydrogenase is a cytosolic enzyme that is released into the perfusate following cell death. LDH expression is ubiquitous due to its central role in anaerobic respiration, where it catalyzes the conversion of lactate to pyruvate. If it is measured in the perfusion fluid, LDH is thus indicative of cell lysis, and so has been used as a marker of pancreas injury [35,36]. The evolution of the LDH concentration in the perfusate allows assessment of the degree of pancreatic injury.

## 8. Exocrine Pancreas Assessment by Non-Invasive Techniques

### Perfusate Amylase and Lipase

Amylase and lipase levels, in perfusion fluid, are key markers in the evaluation of pancreatic suffering during perfusion. Indeed, one of the limitations of pancreas perfusion is the circulation of exocrine proteolytic enzyme in the perfusion fluid. The evaluation of the rate of these parameters is therefore of paramount importance. Barlow et al. and others demonstrated significant rises in the perfusate levels of amylase and lipase during normothermic pancreas perfusion.

## 9. Endocrine Pancreas Assessment by Invasive Techniques

### 9.1. Insulin, Glucagon and Somatostatin

Immunohistochemical analysis allows us to evaluate the effects of perfusion on endocrine pancreas. Thus, glucagon, insulin and somatostatin can be assessed by either immunohistochemistry in tissue biopsies or following release into the perfusate, at regular intervals during the perfusion, which provides information on the viability of the islets of Langerhans throughout the perfusion.

### 9.2. Cell Free DNA of Beta Cell Origin

We have discussed the use of both total cfDNA and a derived integrity index above as markers of cell death above. However, we can use the epigenome to infer the cell type of origin: specifically, through DNA methylation in the context of cytosine–guanine dinucleotides (CpG). This is of particular interest during pancreas preservation and transplantation, where we are specifically interested in beta cell function to improve blood glucose homeostasis in the transplant recipient. There are currently a number of different approaches for cell type inference from cfDNA [79]. Among the most promising is that of Dor and colleagues [80]. The insulin gene promotor contains six CpG sites that are specifically unmethylated in beta cells to allow transcription of the insulin gene, and thus insulin production, but are methylated elsewhere. This can then be measured by either next-generation sequencing or DNA microarray-based methods following bisulfite treatment. Each CpG site was found to be unmethylated in 90–95% of DNA from beta cells, and in 5–15% of DNA molecules from other tissues, which reflects the stochasticity of DNA methylation. However, using a combination of all six CpG sites has been reported to produce a false positive rate of less than 0.01%. We eagerly await the first publications to use such assays to specifically measure beta cell death from a “liquid biopsy” of perfusate during pancreas preservation.

## 10. MRI and MRS

Magnetic resonance imaging (MRI) and spectroscopy (MRS) are non-invasive, non-ionizing techniques that are routinely used to diagnose pathology in vivo.

Application of these techniques to the pancreas enables diagnosis of pre-existing dysfunction, such as pancreatitis [81]. Moreover, these technologies allow a quantification of pancreatic steatosis and fibrosis [82], as well as differentiation of chronic pancreatitis and pancreatic carcinoma in patients [83]. Exploiting these techniques for assessment of ex vivo pancreases holds real promise. For example, Weis et al. used proton (^1^H) MRS to measure the concentration of intracellular lipid of non-adipose pancreatic cells and a total fat concentration within ex vivo pancreases undergoing static cold storage [84]. Pancreatic intracellular lipid concentrations may be a particularly interesting marker of viability given β-cell lipotoxicity [85].

Furthermore, in the context of preclinical research, MRI and MRS technology can determine a temperature in a defined deep volume [86] and allows us to determine pH. Indeed, MRS may measure pH directly with the aid of tracers [87], in which case careful comparison should be made to the point of neutrality at the relevant temperature. Moreover, MRS also measures pH without tracers, using non-invasive methods (e.g., ^31^P-MRS) [88].

Tissue oxygen tension can be evaluated by MRS, using perfluorocarbon probes, with subsequent imaging by measuring the 19F spin-lattice relaxation time [89]. Moreover, phosphorus (^31^P) MRS enables non-invasive measurement of high energy phosphate metabolite concentrations, including ATP and P_i_, and calculation of intracellular pH [90,91]. In ex vivo pancreases, ^31^P-MRS has been used in a range of preclinical studies to assess the effectiveness of preservation techniques and predict transplant outcome [45,92,93,94,95]. Recent work by Carlbom et al. demonstrated the feasibility of performing a pre-transplant assessment of pancreas grafts using ^31^P-MRS [96]. Additionally, phosphocreatine levels can be measured by ^31^P-MRS [97].

Furthermore, MRI allows us to evaluate metabolite concentrations. Indeed, MRI has been used to assess the structural and metabolic changes of livers [98,99], kidneys [100,101], and hearts [102,103] during hypothermic and normothermic machine perfusion. This is preferable as a non-invasive method, facilitating the use of repeated measurement throughout preservation, and measurements can be taken in real time. To date, no studies have combined hypothermic or normothermic machine perfusion with MRI assessment in the pancreas. However, as in other organs, the combination of the two technologies could provide a better assessment of metabolic function and even monitor metabolic recovery during interventions. Additional MRI methods, such as dissolution dynamic nuclear polarization, ^13^C spectroscopic imaging, and intravoxel incoherent motion could also be used to image glycolytic metabolism and perfusion, respectively, in pancreases during perfusion, as has been done previously with other perfused organs [98,101] in order to allow assessment of metabolite flux.

All assessment parameters and assessment techniques are summarized in Table 1. In our opinion, the most important pancreas assessment parameters are presented in Table 2.

## 11. Discussion

This review provides a comprehensive assessment of methods to evaluate the pancreas during preservation. These parameters relate not only to injury between retrieval and transplantation, but also to pre-existing injury in the donor. They are therefore likely to be correlated with pancreas function following transplantation. We highlight both existing methods and areas where further development is needed.

There is a clear need to discover and validate markers to evaluate the pancreas during the preservation period. These will have utility first in pre-clinical research as surrogate markers for either short or long-term graft function after transplantation. Agreement across the field on which markers are valid will substantially expedite pre-clinical experiments assessing the effect of novel strategies and therapeutics for organ preservation.

If valid predictors of transplant outcome during perfusion can be defined, this will also enable the prediction of organ function prior to transplantation. Current assessment of whether a pancreas is suitable for transplantation relies upon donor scoring systems, such as the pancreas donor risk index, and surgical assessment [104]. However, these systems struggle to reliably identify a ‘cut-off’ at which point a marginal organ is too high risk to transplant [105]. Furthermore, there is a large amount of variation regarding which organs are deemed suitable for transplant between different surgeons. Therefore, additional objective measures such as biomarkers are needed to enable increased utilization of marginal organs without compromising patient safety. This problem is compounded by changes over that last decades that have resulted in an increasing disparity between organ supply and demand and have led to pancreases being retrieved from donation after circulatory death (DCD) and extended criteria donors (ECD). These organs are often, but not always, of poorer quality than those from conventional donors, which is exacerbated by the fact that they are more sensitive to both warm and cold ischemia [106]. As well as the use of perfusion strategies to better preserve these pancreases, if we can adequately predict their function following transplant, this will vastly reduce the number of organs that are wasted.

The parameters that we review herein provide some elements of strategies to predict pancreas viability. These may be categorized for different uses based upon a number of criteria. A number of techniques described here are invasive measures, which, whilst suitable for experimental work, are strongly undesirable for clinical use. This is driven by hesitancy to biopsy a pancreas graft, due to risks of complications including pancreatic fistula formation [107]. Similarly, for use in clinical assessment measurement needs to occur in real time rather than requiring lengthy procedures. Assessment must be also considered safe when administering substances such as MRS probes, as they need to be delivered directly to the organ to enable measurement. In all cases, however, direct measurement of indices is preferred.

In our opinion, perfusion dynamics (flow, pressure, vascular resistance), oedema assessment (impedance analysis and wet-to-dry weight), metabolic assessment (pH assessment, whole organ oxygen consumption, tissue oxygen tension, ATP assessment, tissue metabolite concentration and perfusate metabolic concentrations), cell death evaluation (histology, electron microscopy, total cell free DNA and caspase activation), exocrine pancreas assessment, and endocrine pancreas assessment are the most revealing assessment tools, as seen in Table 2.

We note that there is a greater likelihood of achieving good predictive accuracy for graft function following transplantation with a multiparameter approach that combines several different indices. The pancreas is an organ that performs multiple functions, and so by combining a range of markers, it will be possible to predict not only beta cell viability but also possible pathways of graft dysfunction such as post-transplant pancreatitis that is often caused by microvascular dysfunction and subsequent graft thrombosis. These may be selected in either a hypothesis-driven or unbiased manner. In either case, biomarker discovery is hampered by the frequent use of small datasets that result in small domains of validity. An international collaborative approach between multiple laboratories will expedite this research, allowing translation into routine clinical practice much sooner.

## 12. Conclusions

This review evaluated the elements of pancreas assessment during ex vivo machine perfusion, both for assessment in pre-clinical experimental models as well for future use for whole-organ transplantation. Thus, pancreas perfusion is emerging as a helpful tool in the evaluation of pancreases prior to transplantation.

## Figures and Tables

**Table 1:** Assessment parameters and techniques.

Assessment Parameters	Assessment Techniques	Invasive or Non-Invasive
Perfusion dynamics	Flow	Non-invasive
	Pressure	
	Vascular resistance	
Oedema assessment	Macroscopic appearance	Non-invasive
	Weight	Non-invasive
	Impedance analysis	Non-invasive
	Wet-to-dry weight	Invasive
Deep tissue temperature	Transducer	Non-invasive
	B-mode ultrasound	
	Microwave radiometry	
	MRI	
pH	Blood gas analysis (in NMP)	Non-invasive
	MRI	
Metabolic status	ATP	Invasive
	Tissue metabolite concentration by biopsies	Invasive
	Mitochondrial respiration	Invasive
	Tissue oxygen tension using probe	Invasive
	Tissue metabolite concentration by MRI	Non-invasive
	Whole-organ oxygen consumption	Non-invasive
	Perfusate metabolic concentrations (lactates)	Non-invasive
	Tissue oxygen tension using MRS or EPR	Non-invasive
Cell death assessment	Cellular architecture by histology	Invasive
	Ultrastructure by electron microscopy	Invasive
	Total cell free DNA	Invasive
	Caspase activation	Invasive
	LDH	Non-invasive
Exocrine pancreas assessment	Amylase and Lipase in perfusate	Non-invasive
Endocrine pancreas assessment	Insulin, glucagon and somatostatin by immunohistochemistry	Invasive
	Insulin, glucagon and somatostatin in solution perfusion	Non-invasive
	Cell free DNA of Beta cell origin	Invasive

**Table 2:** Pancreas assessment parameters required.

Assessment Parameters	Assessment Techniques
Perfusion dynamics	Flow
	Pressure
	Vascular resistance
Oedema assessment	Impedance analysis
	Wet-to-dry ratio
Deep tissue temperature	Transducer
	B-mode ultrasound
pH	Blood gas analysis (in NMP)
Metabolic status	ATP
	Tissue metabolite concentration
	Tissue oxygen tension using probe
	Whole-organ oxygen consumption
	Perfusate metabolic concentrations (lactates)
Cell death assessment	Cellular architecture by histology
	Ultrastructure by electron microscopy
	Total cell free DNA
	Caspase activation
Exocrine pancreas assessment	Amylase and Lipase in perfusate
Endocrine pancreas assessment	Insulin, glucagon and somatostatin by immunohistochemistry
	Cell free DNA of Beta cell origin
